# Non-invasive estimation of mean pulmonary artery pressure by cardiovascular magnetic resonance in under 2 min scan time

**DOI:** 10.1093/ehjimp/qyad014

**Published:** 2023-07-31

**Authors:** Goran Abdula, Joao G Ramos, David Marlevi, Alexander Fyrdahl, Henrik Engblom, Peder Sörensson, Daniel Giese, Ning Jin, Andreas Sigfridsson, Martin Ugander

**Affiliations:** Department of Clinical Physiology, Karolinska University Hospital, and Karolinska Institutet, Stockholm, Sweden; Department of Clinical Physiology, Karolinska University Hospital, and Karolinska Institutet, Stockholm, Sweden; Department of Clinical Physiology, Karolinska University Hospital, and Karolinska Institutet, Stockholm, Sweden; Institute for Medical Engineering and Science, Massachusetts Institute of Technology, Cambridge, MA, USA; Department of Clinical Physiology, Karolinska University Hospital, and Karolinska Institutet, Stockholm, Sweden; Department of Clinical Physiology, Karolinska University Hospital, and Karolinska Institutet, Stockholm, Sweden; Department of Cardiology, Karolinska University Hospital, and Karolinska Institutet, Stockholm, Sweden; Magnetic Resonance, Siemens Healthcare, GmbH, Erlangen, Germany; Cardiovascular MR R&D, Siemens Medical Solutions USA, Inc, Cleveland, OH, USA; Department of Clinical Physiology, Karolinska University Hospital, and Karolinska Institutet, Stockholm, Sweden; Kolling Institute, Royal North Shore Hospital, and University of Sydney, Kolling Building, Level 12, St Leonards, Sydney, NSW 2065, Australia

**Keywords:** pulmonary hypertension, magnetic resonance imaging (MRI), four-dimensional flow, accelerated imaging

## Abstract

**Aims:**

Non-invasive estimation of mean pulmonary artery pressure (mPAP) by cardiovascular magnetic resonance (CMR) four-dimensional (4D) flow analysis has shown excellent agreement with invasive right heart catheterization. However, clinical application is limited by relatively long scan times. Therefore, the aim of this study was to evaluate the accuracy and time reduction of compressed sensing (CS) accelerated acquisition for mPAP estimation.

**Methods and results:**

Patients (*n* = 51) referred for clinical CMR at 1.5 T or 3 T underwent imaging with both a prototype CS-accelerated and a non-CS-accelerated flow sequence acquiring time-resolved multiple 2D slice phase-contrast three-directional velocity-encoded images covering the pulmonary artery. Prototype software was used for the blinded analysis of pulmonary artery (PA) vortex duration to estimate mPAP as previously validated. CS-accelerated and non-CS-accelerated acquisition showed increased mPAP in 22/51 (43%) and 24/51 (47%) patients, respectively. The mean bias for estimating mPAP between the two methods was 0.1 ± 1.9 mmHg and the intraclass correlation coefficient was 0.97 (95% confidence interval 0.94–0.98). Effective scan time was lower for the CS-accelerated acquisition (1 min 55 s ± 27 s vs. 9 min 6 s ± 2 min 20 s, *P* < 0.001, 79% reduction).

**Conclusions:**

CS-accelerated CMR acquisition enables preserved accuracy for estimating mPAP compared to a non-CS-accelerated sequence, allowing for an average scan time of less than 2 min. CS-acceleration thereby increases the clinical utility of CMR 4D flow analysis to estimate mPAP.

## Graphical Abstract

**Graphical abstract:**
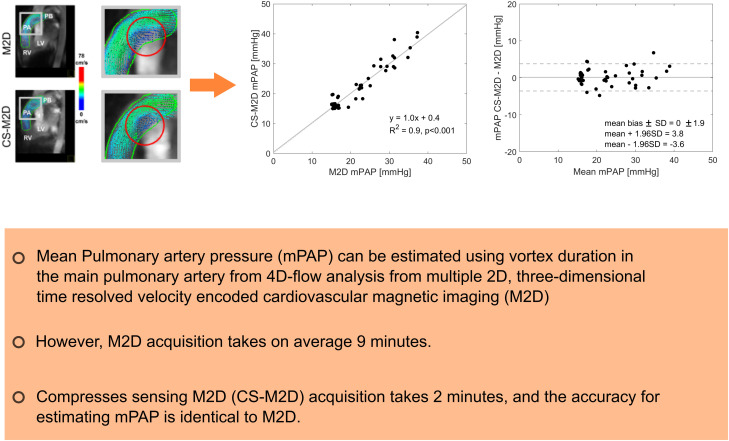
Accelerated flow acquisition for non-invasive estimation of mPAP. Accelerated flow acquisition for 4D flow analysis using compressed sensing technique enables accurate assessment of mean pulmonary artery pressure (mPAP). However, with the important advantage of being faster, which enables the use of CMR 4D flow analysis to determine mPAP and diagnosis of pulmonary hypertension non-invasively. M2D—multi-planar 2D, CS-M2D—compressed sensing multi-planar 2D, RV—right ventricle, LV—left ventricle, PA—main pulmonary artery, PB—pulmonary bifurcation. mPAP—mean pulmonary artery pressure.

## Introduction

Pulmonary hypertension (PH) is a progressive disease associated with high mortality regardless of the underlying pathophysiological aetiology.^1^ PH also has a dominant effect on healthcare worldwide, with about 1% of the global population, up to 10% of individuals aged over 65 years, and at least 50% of patients with heart failure all suffering from PH.^2^ To date, diagnostic evaluation of PH is determined by invasive right heart catheterization (RHC), defined by a mean pulmonary artery pressure (mPAP) of 20 mmHg or greater at rest.^3^ However, the invasive nature of RHC procedures limits applicability, as well as use for early diagnostic screening of PH. Instead, non-invasive imaging, and cardiovascular magnetic resonance (CMR) in particular, offers an important role in the clinical management of patients with PH,^4^ providing an accurate and reproducible assessment of left ventricular (LV) and right ventricular (RV) volumes, function, and mass. CMR 4D flow analysis has also been proposed as a potential non-invasive tool for estimating mPAP,^5,6^ empirically relating the persistence of a pathological flow vortex in the main pulmonary artery (PA) to mPAP. Within this space, 4D flow analysis has shown excellent agreement with invasive RHC and superior diagnostic accuracy as compared to Doppler echocardiography for estimating mPAP.^7^ However, clinical application of the method is limited in part by relatively long scan times, with previously utilized protocols requiring approximately 10 min of acquisition for complete flow mapping of the main PA. Current developments in acceleration techniques such as compressed sensing (CS) are promising and can potentially reduce scan times. However, the accuracy of 4D flow analysis for CS-accelerated compared to non-CS-accelerated acquisition is not known. Therefore, the aim of this study was to perform a head-to-head comparison of CS-accelerated and non-CS-accelerated flow acquisition, assessing discrepancies in estimated mPAP as well as effective scan time.

## Methods

### Patient population

To evaluate the differences between flow sequences with regards to non-invasive mPAP estimation, a prospective study protocol was defined. Between May 2020 and January 2021, *n* = 55 consecutive patients referred for clinical CMR were enrolled in this study. The cohort size was based on power calculations seeking to infer a possible difference of 6% in PA vortex duration between sequences, using a previously known mean vortex duration of approximately 10 ± 10% in an average clinical population. Sample size calculation for α = 0.05, β = 0.20, and power of 0.80 was *n* = 44. Inclusion criteria included patients referred for clinical CMR on a diagnostic basis. Exclusion criteria included known contraindications for CMR as well as arrhythmia, valve prosthesis, or known pulmonary valvular disease. The study was approved by the Stockholm Regional Board of ethics committee, and written informed consent was obtained from each patient before enrolment in the study (EPN: 2011/1077-31/3).

### CMR protocol

CMR was performed at either 1.5 T (*n* = 21) or 3 T (*n* = 34) (MAGNETOM Aera or Skyra, Siemens Healthcare, Erlangen, Germany) using phased array receiver coils with electrocardiography (ECG) gating. As part of the clinical routine, the examination included standard breath-hold cine imaging with steady-state free precession in the short axis and the standard long axis planes. Haemodynamic flow mapping was acquired by positioning a stack of 6–10 gapless slices covering the main PA and the RV outflow tract using multiple 2D slices of ECG-gated time-resolved phase contrast with three-directional velocity encoding, with either GeneRalized autocalibrating partial parallel acquisition (GRAPPA) acceleration (R = 2) with 22 integrated calibration lines, denoted M2D, or CS-acceleration with dual-density phase-encoding using a prototype sequence, denoted compressed sensing multi-planar 2D (CS-M2D). The undersampling factor was R = 3 in the central region and R = 11 in the peripheral region, resulting in an effective acceleration factor of R = 7.7. The relevant parameters for the CS reconstruction were as follows; 40 iterations, spatial regularization factor $\lambda_{s}=0.0003$ and temporal regularization factor $\lambda_{t}=0.001$.

The two phase-contrast sequences were acquired in direct succession using identical geometry and free breathing. Common parameters for both sequences were velocity encoding direction 90 cm/s in all three spatial directions, three-fold averaging to suppress breathing artefacts, in-plane field of view 340 × 276 mm^2^, k-space matrix size 192 × 138 corresponding to an acquired in-plane pixel size of 1.8 × 2.0 mm^2^, slice thickness 6 mm, bandwidth 450 Hz/px, flip angle 15°. Both sequences used retrospective cardiac gating, reconstructing to 20 interpolated time frames.

M2D was acquired using TE (echo time)/TR (repetition time) 4.1/6.4 ms, resulting in an acquired temporal resolution of 77 ms. CS-M2D was acquired using TE/TR 4.1/6.3 ms, resulting in an acquired temporal resolution of 75 ms.

### CMR image processing and image-based mPAP estimation

**Figure 1:**
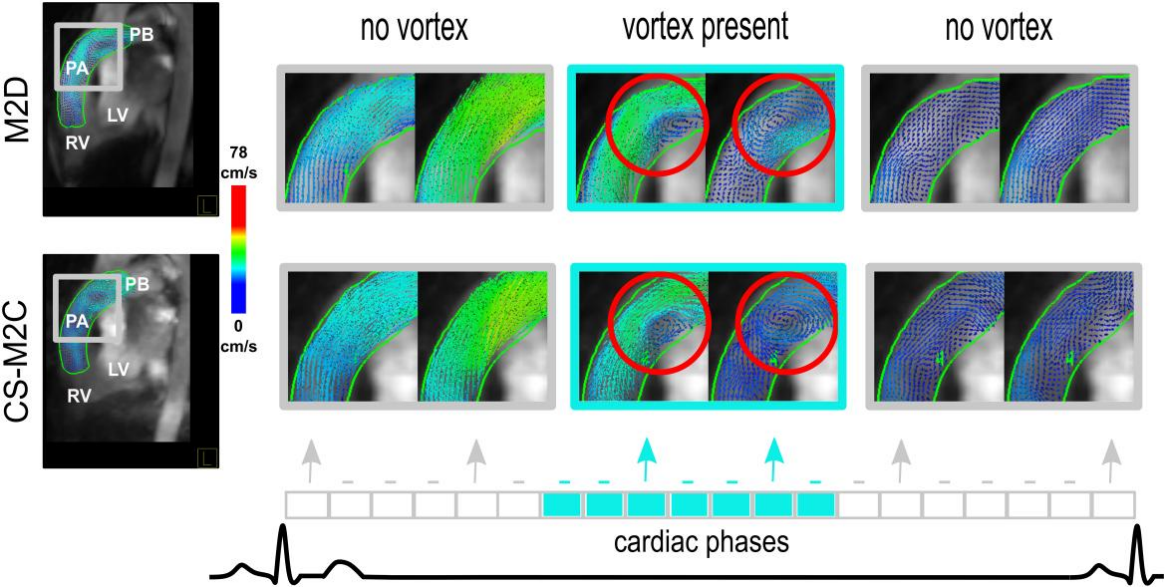
Matched images from selected time frames from a representative patient showing a velocity vector field multi-planar reformat using M2D (top panel) and CS-M2D (bottom panel) multiple 2D slice three-directional time-resolved phase-contrast velocity-encoded sequences, respectively. Empty boxes (grey outline) represent successive time frames in the cardiac cycle. The shaded boxes (light blue) represent the time frames in which a vortex could be visualized. M2D, multi-planar 2D; CS-M2D, compressed sensing multi-planar 2D; RV, right ventricle; LV, left ventricle; PA, main pulmonary artery; PB, pulmonary bifurcation.

All flow datasets were de-identified and analysed using prototype software (4D Flow, Siemens Healthcare, Erlangen, Germany). A fully automatic pre-processing step was applied that performed eddy current compensation and phase-unwrapping as needed. The RV outflow tract and PA were manually segmented to capture the primary flow features in these areas. Streamlines were utilized to qualitatively visualize the presence of potential PA vortices. For quantitative assessment, vector visualization was subsequently used while seeding in 2D views at full reconstructed voxel resolution in order to detect flow vortices in the main PA, following methodologies described in previous work.^8^ Briefly, the presence of a vortex was determined by identifying closed concentric rings in the 4D velocity vector field within the main PA. Vortex presence was expressed in terms of a number of time frames where a vortex could be detected and converted into percentages out of the total number of frames in the cardiac cycle. The complete process is illustrated in *Figure 1*. Mean pulmonary arterial pressure (mPAP) was then estimated using a previously described^9^ empirically determined equation stating:

$$\mathrm{mPA}P_{\mathrm{CMR}}[\mathrm{mmHg}]=\;t_{\mathrm{vortex}}[\text{\%}]+25.44/1.59$$

The 4D flow analysis was performed by two independent readers (GA and JGR, the first and second authors of this paper). The analysis was performed interchangeably on both M2D and CS-M2D datasets in a randomized fashion.

### Statistical analysis

Statistical testing was performed using freely available analysis software (RStudio 1.4, Boston, MA, USA). Continuous variables were expressed as mean ± standard deviation (SD) and categorical variables were presented as percentages. Differences in mean mPAP between CS-M2D and M2D estimations were analysed by student’s *t*-test. The Wilcoxon signed-rank test was used to compare the scan time duration for M2D and CS-M2D. The relationships between mPAP derived from M2D vs. CS-M2D were investigated using the Spearman rank correlation coefficient and Bland–Altman analysis. Likewise, regression analysis was performed to quantify differences in mPAP estimation using CS-M2D and M2D across measurements. Possible bias between field strengths (1.5 T vs. 3 T) was analysed using an unpaired *t*-test. Furthermore, interobserver variability was evaluated by performing Bland–Altman analysis and calculating the intraclass correlation coefficient (ICC) and 95% confidence interval (95%CI) of a randomly selected set of *n* = 20 patients. A *P*-value < 0.05 was considered statistically significant.

## Results

A total of 55 patients were consecutively recruited and four patients were excluded due to poor image quality (two excluded based on the CS-M2D, one based on the M2D, and one due to unrecoverable phase aliasing). As such, 51 patients were included with both CS-M2D and M2D images successfully acquired, with data eligible for 4D flow analysis and estimation of mPAP. Out of the 51 patients included in this study, 34 of them (67%) were found to have underlying left-sides heart disease identified by CMR. Within this subset, 18 patients (53%) exhibited elevated mPAP using M2D. The remaining 17 patients who had normal CMR findings (normal left and RV morphology and function, no late gadolinium enhancement), five patients (29%) had increased mPAP. Patient demographics and clinical data of the study population are summarized in *Table 1*.

**Table 1:** Summary of patient’s demographics and clinical characteristics

Patients	PH *n* = 24	Non-PH *n* = 27	*P*-value
Age, years	49 ± 17	49 ± 19	0.974
Female sex, %	33%	22%	0.666
BSA, m^2^	1.95 ± 0.25	1.93 ± 0.20	0.799
HR, bpm	67 ± 14	68 ± 10	0.975
Clinical diagnosis			
IHD, *n* (%)	2 (8)	5 (19)	0.659
CMP, *n* (%)	6 (25)	6 (22)	0.964
VHD, *n* (%)	3 (13)	1 (0.4)	0.770
PH, *n* (%)	3 (13)	0 (0)	0.709
Myocarditis, *n* (%)	4 (17)	4 (15)	0.969
Normal CMR, *n* (%)	6 (2)	11 (41)	0.844

bpm, beats per minute; BSA, body surface area; CMP, cardiomyopathy; CMR, cardiac magnetic resonance; HR, heart rate; IHD, ischaemic heart disease; PH, pulmonary hypertension; VHD, valvular heart disease.

### Diagnostic differentiation of pulmonary hypertension

Out of the total of 51 patients, analysis by M2D indicated increased mPAP (>20 mmHg) in 24 (47%) patients, and normal evaluated mPAP (≤20 mmHg) in 27 (53%) patients. By comparison, CS-M2D identified 22 (43%) patients with increased, and 29 (57%) patients with normal mPAP, respectively. As such, discrepancies between M2D and CS-M2D in diagnostic differentiation were observed in two patients. Note that in these patients, both estimates were very close to the clinical threshold (mPAP = 22 mmHg by M2D vs. mPAP = 19 mmHg by CS-M2D). Furthermore, in the patient group with mPAP < 20 mmHg by M2D (*n* = 27), 22 of these exhibited no visible vortex in any of the flow sequences, 4 patients had a pathological vortex formation in the main PA identified only by CS-M2D, and one patient had pathological vortex formation in the main PA identified only by M2D.

### Quantitative mPAP estimation

**Figure 2:**
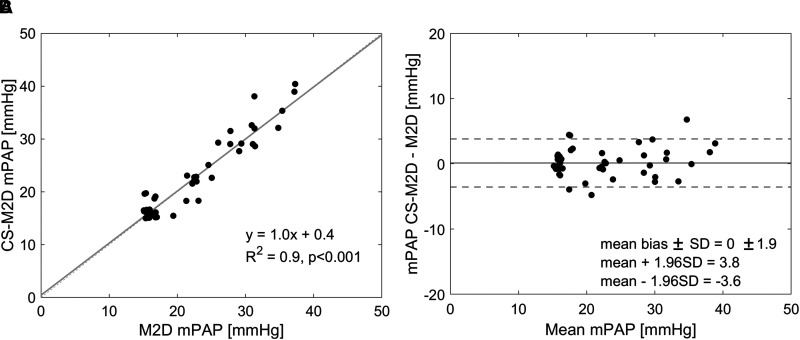
(*A*) Linear regression (solid line) of estimated mean PA pressure (mPAP) by compressed sensing (CS)-accelerated and conventional 4D flow analysis in all analysed patients. The dotted line shows a line of identity. (*B*) Bland–Altman plot of estimated mPAP by 2D conventional and CS CMR flow in patients with both observable vortex by 4D flow analysis. Mean ± SD bias was 0.1 ± 1.9 mmHg. Note that data jitter is introduced to improve the visibility of overlapping data points. M2D, multi-planar 2D; CS-M2D, compressed sensing multi-planar 2D.

Overall, estimation of mPAP between M2D and CS-M2D did not differ (21.7 ± 7.0 vs. 21.9 ± 7.2 mmHg, *P* = 0.641). In particular, as shown in *Figure 2A*, mPAP estimated by CS-M2D revealed excellent agreement with estimates obtained by M2D (R^2^ = 0.93, *P* = 0.001), and with negligible bias (mean bias 0.2 ± 2.3 mmHg at 1.5 T (*n* = 20), 0.1 ± 1.5 mmHg at 3 T; overall mean bias 0.1 ± 1.9 mmHg (*Figure 2B*).

### Effective scan time

Across all subjects, scan time was 9 min 6 s ± 2 min 20 s for M2D compared to 1 min 55 s ± 27 s for CS-M2D, representing a reduction of 79% in effective scan time (*P* < 0.001).

### Interobserver variability

**Figure 3:**
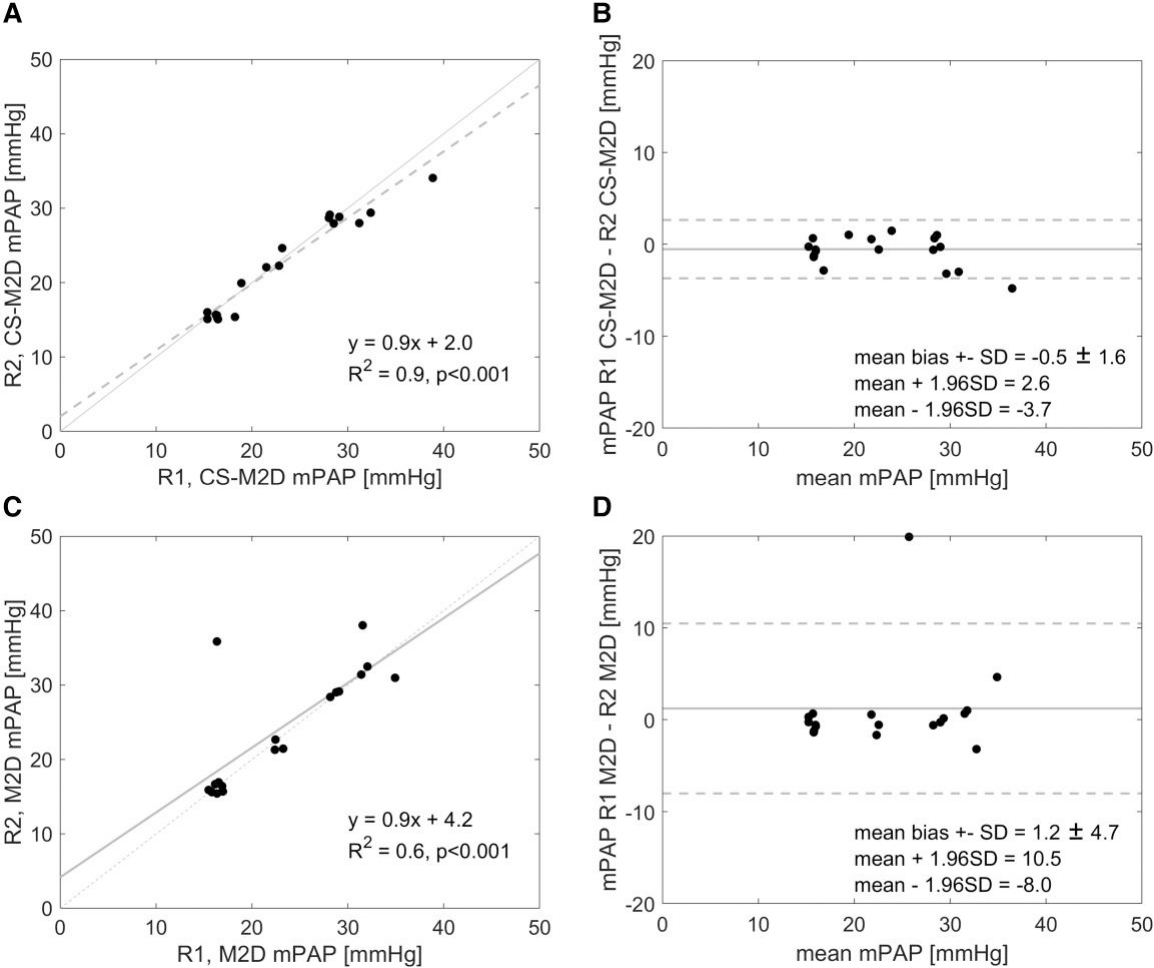
Linear regression and Bland–Altman plots of mean PA pressure (mPAP) estimated by Reader 1 (R1) and Reader 2 (R2) by CS-M2D) (*A*, *B*) and multi-planar 2D (M2D) (*C*, *D*). Note that data jitter is introduced to improve the visibility of overlapping data points.

From the interobserver variability analysis, good agreement was observed with the ICC for the estimation of mPAP being 0.97 (95%CI 0.94–0.98). Similarly, the overall interobserver variability yielded a good agreement for mPAP by both the M2D and CS-M2D, with ICC of 0.95 (95%CI 0.89–0.98) for estimated mPAP. As shown in *Figure 3*, high linear regression coefficients and low mean bias were also all reported for interobserver variability analysis on both CS-M2D and M2D analysis, respectively.

## Discussion

The main finding of the study is that CS-accelerated CMR acquisition enables preserved accuracy for estimating mPAP compared to a non-CS-accelerated sequence, allowing for an average scan time of less than 2 min. This represents a substantial improvement opening up for non-invasive mPAP estimation without extensively added acquisition duration for existing clinical protocols. The clinical potential of CS-M2D is further emphasized by the minimal bias observed between different field strengths (1.5 T vs. 3.0 T) and different clinical readers.

In this study, the estimation of mPAP from phase-contrast flow images was based on the empirical regression model established by Reiter *et al.*,^9^ with image-derived vortex duration shown to carry high diagnostic accuracy for the estimation of pathological mPAP exceeding 16 mmHg. These results were also corroborated by a recent study in which M2D exhibited excellent agreement with invasively measured mPAP (*n* = 40) and superior diagnostic performance for detecting PH compared to Doppler echocardiography.^7^ The results of the current study represent a continuation towards routine clinical implementation, incorporating sequence acceleration through CS protocols, with resulting scan time efficiency and maintained accuracy that pushes the utility towards a more streamlined clinical workflow. The majority of the patients in the current study population had left-sided heart disease as the aetiology underlying the PH. Consequently, the application of the CS-M2D technique to other aetiologies for PH remains to be studied. Notably, the M2D technique has been accurately applied across all subtypes of PH aetiologies,^9^ and there is no known reason in principle why CS-M2D would differ in performance compared to M2D in other PH subgroups. Within the setting of PH, RHC is regarded as the reference standard for assessing mPAP and establishing the diagnosis. However, the invasiveness, complication risk,^10^ and relatively long procedure time associated with percutaneous catheterization limit its use for screening purposes and may delay diagnosis.^11^ Instead, Doppler echocardiography remains the primary non-invasive alternative technique used to assess patients with suspected PH, where a simplified assessment of the apparent haemodynamic environment is used to estimate peak systolic pulmonary pressure.^12^ However, echocardiographic assessment of pulmonary pressure has several limitations including limitations in acoustic window^13^ and accuracy,^14^ as well as a modest correlation between systolic PA and mPAP.^15^ Moreover, almost half of all patients being referred for RHC lack a detectable tricuspid regurgitation jet by Doppler imaging,^16^ effectively reducing the applicability of echocardiography. As a result, there is still a clear clinical need for alternative and precise non-invasive methods for the clinical estimation of mPAP, moving beyond surrogate estimates from RV structure and function. In a recent study, the vortex duration in the main PA showed a strong correlation with total arterial compliance (R^2^ = 0.72; *P* < 0.001), a moderate correlation with pulmonary vascular resistance (R^2^ = 0.47; *P* < 0.001), a weak correlation with pulmonary wedge pressure, RV volume index, and tricuspid annular plane systolic excursion (R^2^ = 0.08–0.15, *P* < 0.05), and no correlation with RHC cardiac output, RV ejection fraction and heart rate (*P* = 0.06–0.38). Notably, CMR vortex duration was the singular measure that was most closely associated with mPAP (R^2^ = 0.85; *P* < 0.001).^7^ As shown in the current study, CS-accelerated phase-contrast CMR could represent such a tool, effectively enabling accurate mPAP estimation without any substantial addition in protocol time. CMR adds incremental diagnostic value for patients with suspected PH, with standardized sequences allowing for simultaneous quantification of LV and RV function, myocardial fibrosis, or other underlying conditions, all in the very same imaging session.^17^ In the clinical setting, CMR shortens the time delay for PH diagnosis and treatment which has the potential to improve prognosis in PH.

### Limitation

There are some limitations that should be acknowledged. A relatively small study population was utilized. Although sufficiently powered to identify the expected differences in mPAP between the compared sequences, this does not mean that the current data necessarily can be generalized to a wider spectrum of cardiac conditions. Specifically, we chose to exclude patients with arrhythmia and severe pulmonary valvular disease, since these conditions are known to interfere with phase-contrast CMR data quality. The ability to non-invasively estimate mPAP by CMR in these patients—and in particular the difference between M2D and CS-M2D—would thus have to be assessed in separate, future analysis. Furthermore, left-sided heart disease, including left-sided valvular heart disease (VHD) is frequently the underlying aetiology of PH, and by far represents the largest application of this technique. As such our population is appropriately chosen. Conditions such as frequent arrhythmia and pulmonic valve disease were excluded from this study since they can interfere with phase-contrast CMR data quality in the main PA. Further studies are justified to evaluate the utility of CMR vortex duration measurement for the estimation of mPAP in such populations. Our study utilized intramodality validation, with the non-CS-accelerated M2D sequence used as a reference for the CS-M2D equivalent. This was performed in light of previous work successfully validating M2D analysis against invasive catheterization at multiple institutions.^5,6^

This study used a proprietary software tool for image analysis (4DFlow, Siemens Healthcare, Erlangen, Germany). This choice was necessitated by the need to perform multi-planar reconstruction of velocity vector fields in order to reliably visualize and identify vortex durations. To the best of our knowledge, only this tool offers this functionality at this time. Further investigations are justified to determine if the same approach can be replicated in other analysis tools that may offer such functionality moving forward.

In this cohort of patients consecutively referred for CMR were used, invasive RHC was not available. Previous studies using non-CS-accelerated CMR 4D flow analysis demonstrated excellent association with invasively (RHC) obtained mPAP.^7,9,18^ Furthermore, non-CS-accelerated CMR 4D flow analysis has been shown to have good agreement compared to echocardiography regarding the estimation of mPAP.^5,19^ Nevertheless, a head-to-head comparison between CS-accelerated CMR and invasive RHC could be used to corroborate the findings of this study. Although we reported a sizeable reduction in acquisition time for CS-M2D vs. M2D, this did not include the time required for post-processing and quantitative estimation of mPAP between the two methods. However, although minor qualitative differences were indicated between sequences by the clinical readers, the time required to manually assess vortex presence was identical between the two sequences, especially when considering the same patient and scan session (data not shown). In this study, none of the patients had an mPAP exceeding 40 mmHg. Among patients with underlying cardiac disease and PH, left-sided heart disease accounts for 65–80% of cases.^20^ Consequently, the results for this particular group with left-sided heart disease and elevated mPAP between 20–40 mmHg indicate excellent performance. However, it is worth noting that patients with mPAP higher than 40 mmHg have been studied using non-CS 4D flow analysis while maintaining high accuracy.^6^ Therefore, there are no indications that CS-M2D may behave differently at longer vortex duration.

## Conclusions

CS-accelerated CMR flow acquisition for 4D flow analysis exhibits excellent agreement with non-CS-accelerated acquisition for quantitative estimation of mPAP. Further, the CS-accelerated sequence reduced effective scan time to under 2 min and was robust at both acquisition field strengths and inter-reader variations. Consequently, CS-accelerated CMR in the diagnosis of PH shows clinical utility.

## Data Availability

All original data utilized for this work are available upon reasonable request to the corresponding author.
